# The Effects of Kernel Type (Inshell, Shelled and Split Almonds) on the Growth and Aflatoxin Production of *A. flavus* Under Different Combinations of Water Activity and Temperature

**DOI:** 10.3390/toxins16110493

**Published:** 2024-11-16

**Authors:** Barbara Szonyi, Guangwei Huang, Tim Birmingham, Dawit Gizachew

**Affiliations:** 1 Department of Chemistry and Physics, Purdue University Northwest, Hammond, IN 46323, USA; bszonyi@pnw.edu; 2 Almond Board of California, 1150 Ninth St., Ste. 1500, Modesto, CA 95354, USA

**Keywords:** aflatoxins, almonds, *Aspergillus flavus*, mycotoxins, nuts

## Abstract

Almonds are susceptible to infestation by *Aspergillus flavus*, an aflatoxin-producing fungus. The objective of this study was to investigate the effects of kernel type (inshell, shelled and split almonds) on the ability of *A. flavus* to grow and produce aflatoxins at different combinations of temperature (20, 27 and 35 °C), water activity (0.85, 0.92, 0.95 and 0.98 a_w_) and incubation period (10, 20 and 30 days). There was no fungal growth at 0.85 a_w_ on any of the kernel types. At 0.92 a_w_, only the split kernels supported growth and aflatoxin synthesis. The fungus was able to grow and produce aflatoxins on all three kernels at 0.95–0.98 a_w_ and 20–35 °C. At 0.98 a_w_, high total aflatoxin concentrations (>300 µg/kg) were found on the shelled and split kernels at all temperatures. On the inshell nuts, the fungus produced up to 372 µg/kg of total aflatoxins at 0.98 a_w_ and 27 °C. Regression analysis showed that significantly higher levels of aflatoxins were produced at 27 °C (as compared to at 20 and 35 °C) on shelled and split almonds. Incubation time was also a significant predictor of aflatoxin accumulation. The results of this study indicated that shipping almonds below 0.85 a_w_ and reducing storage time would significantly decrease the risk of infestation and aflatoxin production by *A. flavus*.

## 1. Introduction

Aflatoxins are a group of carcinogenic, teratogenic and immunosuppressive fungal metabolites produced by *Aspergillus flavus* and *A. parasiticus*. These ubiquitous fungi colonize a large variety of crops worldwide in the field and after harvest [1]. The four main types of aflatoxins are AFB_1_, AFB_2_, AFG_1_ and AFG_2_. The International Agency for Research on Cancer (IARC) classified all four aflatoxins as highly carcinogenic [2]. Toxigenic strains of *Aspergillus flavus* can produce the most toxic form, AFB_1_, as well as AFB_2_. Currently, strict regulations are in place to protect humans and animals from the consumption of aflatoxin-contaminated food and feed. The European Union set the limits at 8.0 µg/kg for AFB_1_ and 10.0 µg/kg for total aflatoxins in ready-to-eat almonds [3]. The United States Food and Drug Administration (FDA) established the maximum permissible level for total aflatoxins at 20 µg/kg [4]. The low tolerance for aflatoxins is a significant challenge for almond producers worldwide. For example, a recent survey including 200 ready-to-eat nuts in Italy showed that 10% of nuts (almonds and pistachios) contained total aflatoxins higher than the legal limit [5]. Crop rejection due to aflatoxins has also been a serious concern to the California almond industry. In the calendar year 2023, Japan had 29 rejections of California almond shipments for exceeding the aflatoxin limit [6]. Therefore, limiting the growth of aflatoxigenic molds on almonds is essential to reduce economic losses in the almond industry.

Among susceptible crops, almonds (*Prunus amygdalus*) have a moderate to high risk of contamination with aflatoxins [7]. Today, 3.5 billion pounds of almonds are being produced globally. The United States (US) is the largest producer of almonds in the world, representing over 80% of the global almond output. Nearly 100% of US almond production is in the Central Valley of California, with a farmgate value of $4.6 billion in 2022 [8].

*Aspergillus flavus* is the most common *Aspergillus* species naturally infecting almonds in California orchards [9]. Contamination of almonds with *A. flavus* can occur during the development of the fruit in the field, during drying on the orchard soil or during transport and storage under favorable moisture conditions [10]. Aflatoxin production on almonds is influenced by several environmental factors, particularly the water activity (a_w_) of the kernels and temperature [7]. In addition, the navel orangeworm (NOW) insect, *Amyelosis transitella*, is a major contributor to the infestation of almonds by mycotoxigenic fungi and the subsequent accumulation of aflatoxins. A study investigating the effect of the NOW on aflatoxin levels in almonds found that significantly higher levels of aflatoxin contamination occurred in NOW-damaged kernels, compared to in undamaged kernels [11]. In undamaged kernels, the presence of intact shells and/or seed coats are thought to provide protection from invasion by *A. flavus* [12]. Therefore, even under similar environmental conditions, the degree of fungal spoilage and aflatoxin accumulation could be markedly different depending on the kernel type. At the same time, studies that compare the growth and aflatoxin accumulation of *A. flavus* on different types of almond kernels are lacking. This information is essential to almond producers, because different types of almond kernels may need different shipping and storage conditions to prevent economic losses from mold growth and aflatoxin accumulation by *A. flavus*.

Many studies have been conducted on the growth and aflatoxin production of *A. flavus* on almonds in California, with the goal of reducing nut contamination with this fungus [9,12,13,14]. Even so, the problem still persists, partly because aflatoxigenic fungi, such as *A. flavus*, are always present in the litter and/or soil of the almond orchards [9]. During harvest, almonds are shaken from the trees and are subsequently left to dry on the orchard floor, which facilitates their infestation by toxigenic *Aspergillus* spp. [11]. Additionally, the elimination of aflatoxins from contaminated kernels is virtually impossible, due to the extremely heat-stabile nature of aflatoxin molecules [13]. To date, there is no single post-harvest technology that can effectively and safely remove aflatoxins from raw almond kernels [14]. Because of these challenges, it is critically important to control the environmental conditions, especially water activity and temperature, during storage and transport to avoid mycotoxin accumulation on the kernels. Accurate information is needed on the exact range of water activities and temperatures that are conducive to fungal growth and aflatoxin accumulation on almonds. Also, in addition to data on optimal conditions, the limiting conditions for growth and aflatoxin production also need to be identified for the successful implementation of effective risk mitigation strategies.

The goal of the present study was to reduce the existing knowledge gaps and thereby assist almond producers in optimizing post-harvest conditions for different almond kernels. The objective was to investigate the effects of kernel type (inshell, shelled and split almonds) on the ability of *A. flavus* to grow and produce aflatoxins at different combinations of temperature (20, 27 and 35 °C), water activity (0.85, 0.92, 0.95 and 0.98 a_w_) and incubation period (10, 20 and 30 days). These conditions were carefully selected to represent a comprehensive range of water activities and temperatures that might occur during the transport and storage of almonds. The results of this study show the environmental conditions which promote or inhibit growth and aflatoxin production by *A. flavus* on the different almond kernels, thereby assisting with the design of safe shipping and storage conditions for almonds.

## 2. Results

### 2.1. Fungal Growth and Total Aflatoxin Production at 0.85 and 0.92 a_w_

**Figure 1:**
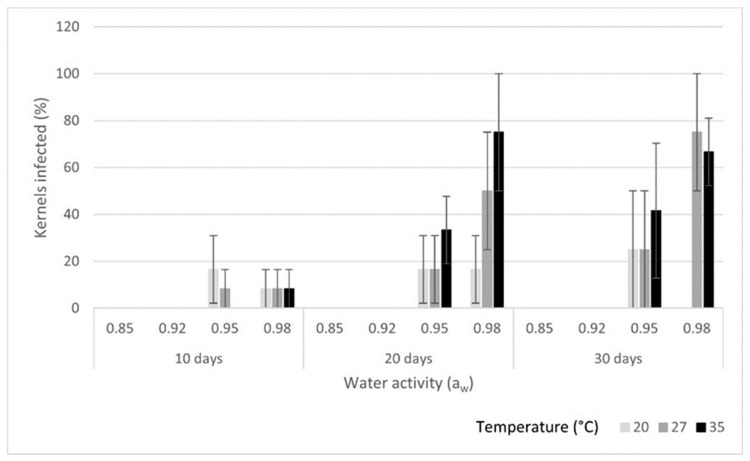
Growth of *A. flavus* on inshell almond kernels at each combination of temperature and water activity. Error bars show standard deviations (SDs).

**Figure 2:**
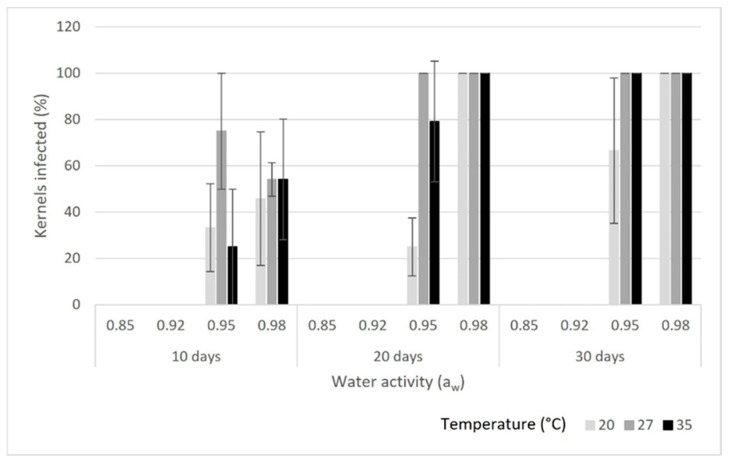
Growth of *A. flavus* on shelled almond kernels at each combination of temperature and water activity. Error bars show standard deviations (SDs).

**Figure 3:**
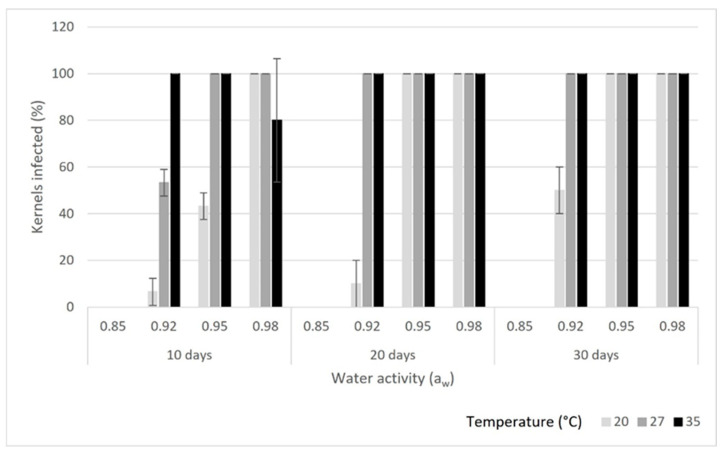
Growth of *A. flavus* on split almond kernels at each combination of temperature and water activity. Error bars show standard deviations (SDs).

Fungal growth was not detected at 0.85 a_w_ on any of the kernel types (Figure 1, Figure 2 and Figure 3) for up to 6 months at the three study temperatures (20, 27 and 35 °C). Also, *A. flavus* did not show any growth at any of the temperatures at 0.92 a_w_ on the inshell and shelled almonds for up to 30 days. On the split kernels, however, *A. flavus* was able to grow and produce aflatoxins at the three temperatures at 0.92 a_w_. The fungus grew slowly at 20 °C on the split almonds, and it only infected 50% of the kernels by day 30. In contrast, *A. flavus* grew rapidly at 35 °C, reaching 100% coverage by day 10 on the split kernels. Aflatoxin production (Table 1) increased with time, reaching high levels by day 30 (>400 µg/kg) at 27 and 35 °C. In comparison, aflatoxin concentration remained lower at 20 °C on the split kernels (70 µg/kg). Thus, at this water activity, low temperature (20 °C) supported slow growth and lower aflatoxin production on the split almonds. Conversely, higher temperatures (27 and 35 °C) combined with 0.92 a_w_ resulted in rapid growth and high levels of aflatoxin by day 30 on the split kernels.

### 2.2. Fungal Growth and Total Aflatoxin Production at 0.95 a_w_

At 0.95 a_w_, *A. flavus* exhibited some growth on inshell, shelled and split almonds at all three temperatures. On the inshell kernels, there was moderate growth (up to 33.3%) at all three temperatures throughout the study period. There was low aflatoxin production (<20 µg/kg) at day 10 at all temperatures on the inshell almonds, and aflatoxin levels remained low at 20 °C throughout the study period. In contrast, aflatoxin concentrations reached high levels at 27 and 35 °C by days 20 and 30 on the inshell almonds. The highest aflatoxin concentration on inshell nuts was 340 µg/kg at 35 °C on day 30. On the shelled kernels, growth reached 100% at 27 and 35 °C, whereas only 66.6% of almonds were infected by day 30 at 20 °C. Aflatoxin production on shelled almonds was high, and it increased over time at all three temperatures. By day 30, aflatoxin levels reached 508 µg/kg at 27 °C and 463 µg/kg at 35 °C. While aflatoxin concentration was lower at 20 °C on the shelled almonds, it reached 133 µg/kg by day 30. On split almonds, there was rapid growth and high aflatoxin production (>200 µg/kg) at all three temperatures. By day 30, aflatoxins levels exceeded 400 µg/kg at 20 and 27 °C on the split kernels. Even though the fungus grew at 0.95 a_w_ on all three kernel types and temperatures, growth was slower and aflatoxin production lower at 20 °C compared to at higher temperatures. On all three kernels, *A. flavus* produced very high levels of aflatoxins at 0.95 a_w_ (>300 µg/kg on inshell and >400 µg/kg on shelled and split nuts) by the end of the study period, indicating that there was an overall tendency for aflatoxin concretions to increase over time at 0.95 a_w_.

### 2.3. Fungal Growth and Total Aflatoxin Production at 0.98 a_w_

Growth and aflatoxin synthesis were both low (<9% and <20 µg/kg, respectively) on the inshell kernels at all three temperatures at day 10. By day 20, there was strong growth (50%) and high aflatoxin production (372 µg/kg) on the inshell kernels at 27 °C and at 35 °C (75% and 362 µg/kg, respectively), while there was low growth and aflatoxin production at 20 °C (16.6% and 3 µg/kg, respectively). At day 30, growth remained high at 27 °C and 35 °C on the inshell kernels, and growth remained low at 20 °C. On the shelled kernels, A. flavus grew well and produced high levels of aflatoxins at 27 °C (343 µg/kg); aflatoxin production was also high at 20 °C (209 µg/kg) and 35 °C (188 µg/kg) on day 10. There was substantial growth on the split kernels at all three temperatures by day 10, with high aflatoxin production at 27 °C (327 µg/kg) and 20 °C (207 µg/kg). By day 20, there was 100% growth and high aflatoxin production (>300 µg/kg) on both the shelled and the split kernels at all three temperatures. On the shelled kernels, both growth (100%) and aflatoxin levels (>290 µg/kg) remained high at all three temperatures at day 30. Similarly, on the split kernels, fungal growth (100%) and aflatoxin levels (>278 µg/kg) remained high at all three study temperatures at day 30.

### 2.4. Statistical Analysis and Linear Regression Models

**Figure 4:**
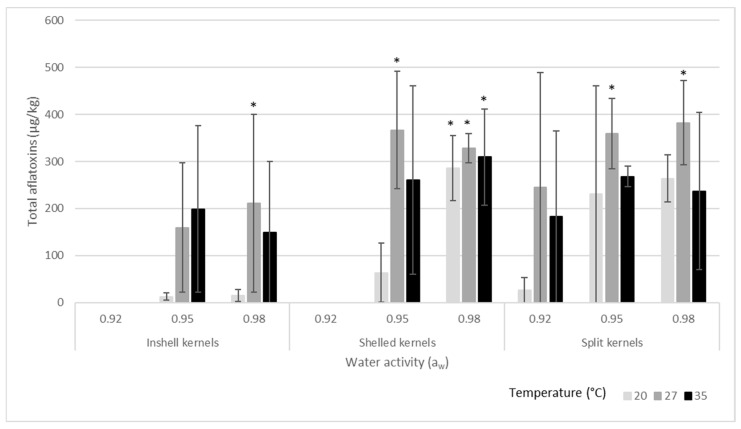
Total aflatoxin (AFB_1_ + AFB_2_) production of *A. flavus* on inshell, shelled and split almond kernels at each combination of temperature and water activity (averaged over 10, 20 and 30 days of incubation). Error bars show standard deviations. Asterisks denote significantly different values within the dataset based on Tukey’s multiple comparison test.

Figure 4 shows statistical comparisons of the total aflatoxin production at the same water activity and temperature conditions averaged over 10, 20 and 30 days of incubation. For example, this figure shows that on the inshell kernels, aflatoxin production was significantly different at 0.98 a_w_ and 27 °C compared to that at 0.98 a_w_ and 20 or 35 °C.

The multivariable linear regression model for inshell kernels (Table 2) revealed that 27 and 35 °C were significantly more favorable to aflatoxin synthesis than 20 °C (*p* < 0.033 and *p* < 0.044, respectively). Furthermore, the model coefficient for 27 °C (114.15) was higher than the coefficient for 35 °C (106.91), indicating that 27 °C was the optimum temperature for aflatoxin synthesis on the inshell almonds. On shelled (Table 3) and split almonds (Table 4), significantly more aflatoxin was produced at 27 °C (*p* < 0.017 and *p* < 0.007, respectively), compared to at 20 and 35 °C. Therefore, 27 °C was the optimal temperature for aflatoxin synthesis on all three kernel types. Regarding the effects of water activity, aflatoxin levels were significantly higher at 0.95 and 0.98 a_w_, compared to at 0.92 a_w_ on inshell, shelled and split almonds. The optimum water activity for aflatoxin production was 0.98 a_w_ for inshell, shelled and split kernels, because their respective model coefficients were higher at 0.98 a_w_ (125.21, 307.85 and 143.01) than at 0.95 a_w_ (123.66, 230.27 and 134.72). The length of incubation was also an important factor in aflatoxin synthesis on all three types of kernels. For the inshell (*p* < 0.043), shelled (*p* < 0.024) and split almonds (*p* < 0.001), longer incubation time resulted in significantly higher levels of aflatoxin.

## 3. Discussion

This study has demonstrated the effect of kernel type on the ability of *A. flavus* to grow and synthesize aflatoxins under various conditions of temperature and water activity on almonds. Split kernels supported the growth and aflatoxin production of *A. flavus* at a wider range of water activities (0.92–0.98 a_w_), but the most favorable water activity for fungal growth and aflatoxin synthesis was similar at 0.98 a_w_ for inshell, shelled and split almonds. Also, the optimum temperature for aflatoxin production was at 27 °C on all kernel types. Furthermore, longer incubation time was another key factor influencing aflatoxin accumulation.

*Aspergillus flavus* has been shown to grow and produce aflatoxins under a wide range of environmental conditions on various nuts, oil seeds and other types of food. Some studies have reported lower water activity requirements for fungal growth than were observed for almonds in the present study. For example, it was revealed that *A. flavus* was able to grow at a range of 0.86–0.98 a_w_ and temperatures of 20–35 °C on both ground Nyjer seeds [15] and ground flax seeds [16]. On ground Nyjer seeds, the optimum conditions for both fungal growth and aflatoxin production for *A. flavus* NRRL 3357 were at the range of 0.90–0.98 a_w_ and temperatures of 27–35 °C. On ground flax seeds, the fungus grew well and produced aflatoxins at 0.90–0.94 a_w_ and 27 °C, as well as at 0.86–0.98 a_w_ and 35 °C. Another study using an *A. flavus* strain isolated from maize in Italy [17] showed that the lowest water activity required for growth on maize was 0.83 a_w_, which is significantly lower than was found in this study for almonds. On split almonds, a minimum of 0.92 a_w_ was required for growth and aflatoxin production by *A. flavus*, and even higher water activity (0.95 a_w_) was needed for growth and aflatoxin synthesis on the inshell and shelled almonds. Similarly, on sorghum grains, the minimum water activity for growth of *A. flavus* was reported to be 0.91 a_w_ [18]. Gallo et al. [7] conducted studies on almond-based media using *A. flavus* ITEM 7828 and found that the fungus did not grow at 0.90–0.93 a_w_ at 20 °C, which concurs with the suppressed growth of *A. flavus* 3357 at 0.92 a_w_ on inshell and shelled almonds. On the other hand, the split almond kernels supported slow growth and low levels of aflatoxin production at 0.92 a_w_ and 20 °C in this study. Additionally, the same study reported that maximum growth and aflatoxin production occurred at 0.96 a_w_ and 28 °C on the almond-based medium, which is close to the optimum conditions encountered in the present study (0.98 a_w_ and 27 °C). Furthermore, there was a marked reduction in aflatoxin production at 20 and 37 °C on an almond-based medium, compared to at 28 °C. Similarly, in our study, the statistical analysis showed significantly higher levels of aflatoxin production at 27 °C as compared to at 20 and 35 °C on the shelled and split almonds. The *A. flavus* strain isolated from maize in Italy [17] had a slightly lower optimum temperature for aflatoxin production at 25 °C. Conversely, high incubation temperature (35 °C) was the most favorable to aflatoxin production on ground flax seeds [16]. Another study in China observed that maximum amounts of AFB_1_ were produced at 33 °C and 0.96 a_w_ by *A. flavus* strain YC-15 on polished rice [19]. On cured-meat-based media, *A. flavus* CBS 573.65 exhibited optimal growth at 25 °C and 0.95 a_w_ [20]. The results of these studies suggest that the minimum water activity level for fungal growth and the optimum temperature for aflatoxin production vary significantly depending on the structure and composition of the substrate as well as the fungal strain. Furthermore, previous works have demonstrated that different strains of *Aspergillus flavus* can show different behaviors depending on environmental conditions. For example, Casquete et al. [21] studied three different *A. flavus* strains and found that maximum aflatoxin production on a cheese-based medium occurred at 0.95 a_w_ and 25 or 30 °C, depending on the strain. In order to further investigate the effect of fungal strain on growth and aflatoxin production on almond kernels, future studies using different strains of *A. flavus* will need to be carried out.

It has been proposed that aflatoxin synthesis can be regarded as a stress response by mycotoxigenic fungi to slightly unfavorable conditions of temperature and water activity. Under such conditions, slow fungal growth might accompany high aflatoxin production [22]. In this study, however, slow growth conditions corresponded with significantly lower aflatoxin production, which was observed at 20 °C. At the same time, consistently high aflatoxin levels were encountered under rapid growth conditions, particularly at 0.98 a_w_ and higher temperatures (27 and 35 °C). *Aspergillus flavus* exhibited similar behavior on ground flax seeds [16], where both rapid growth and high aflatoxin production were observed under the same conditions (0.90–0.94 a_w_ and 27–35 °C).

In addition to the effects of water activity and temperature, statistical analysis indicated a significant positive effect of incubation time on aflatoxin production in the current study. Of the eight aflatoxin measurements that exceeded 400 µg/kg in this study, seven were recorded at day 30 of incubation on shelled and split almonds. This finding shows that over time, very high levels of aflatoxins can be accumulated by *A. flavus* on almonds. A similar conclusion was reached by other investigators [23] who inoculated almonds with a toxigenic *A. flavus* strain and observed that prolonged storage for 18 months significantly increased the aflatoxin contents of the kernels compared to those of shorter storage periods (2–3 months). Consequently, reducing the duration of storage and transit time would lower the risk of aflatoxin accumulation on almonds.

Some of the samples in this study contained very high levels of total aflatoxins. The highest concentration of total aflatoxin was 508 µg/kg on shelled almonds, 486 µg/kg on the split kernels and 372 µg/kg on the inshell nuts. These results clearly show the capacity of *A. flavus* to produce aflatoxin levels far above the legal limits under favorable conditions on almonds.

Fanelli and Fabbri [24] revealed that oil-rich seeds may contain high concentrations of aflatoxins due to lipid-peroxidation-induced aflatoxin synthesis. Particularly unsaturated fatty acids have been shown to stimulate aflatoxin synthesis by mycotoxigenic species such as *A. flavus*. Almonds are very rich in unsaturated fatty acids, with an oil content of 31% oleic and 12% linoleic acids [25], which may contribute to the production of high levels of aflatoxins in this nut.

In spite of the capacity of *A. flavus* to produce high concentrations of aflatoxins on almonds, several field studies discovered low levels of aflatoxin contamination in almonds. For example, surveys of different nuts in Saudi Arabia [26] and Qatar [27] did not find detectable levels of aflatoxins on almonds. A study in Pakistan found total aflatoxin levels in inshell almonds below the EU limit [28]. A study in Portugal including twenty-one almond samples detected 4.97 µg/kg of AFB1 in only one (5%) of the samples analyzed [29]. Climatic conditions, especially low humidity levels and hot temperatures in these survey areas, might have contributed to the low aflatoxin levels. On the other hand, surveys involving peanuts or groundnuts indicated that *Aspergillus flavus* was able to produce extremely high levels of aflatoxins on these nuts, even under field conditions. For example, total aflatoxin concentrations of 3135 µg/kg and 1041 µg/kg have been reported in groundnut samples from markets in Ethiopia [30] and Nigeria [31], respectively, which were attributed to *A. flavus*.

Almond shells and seed coats have been shown to reduce infestation with *A. flavus* [12]. In the present study, shells provided some protection from fungal invasion, as the inshell kernels supported the least amount of growth, even under ideal conditions. The hard almond shells mainly consist of cellulose, hemicellulose and lignin, which do not provide sufficient nutrients for the growth of mycotoxigenic fungi [32]. In contrast, the split kernels, which lack shells and partially expose the nutrient-rich nut meat, supported the fastest growth. The fungus also grew well on the shelled kernels, although longer time was required to reach 100% coverage (>10 days), which suggests that the seed coat (without the shells) did not provide an effective barrier to invasion by *A. flavus*, especially during longer incubation times. The almond skin contains biologically active molecules such as phenolic compounds, which may reduce or slow the growth of molds [33]. It has also been suggested that the presence of shells and seed coat reduced aflatoxin contamination in almonds [11]. In this study, however, A. *flavus* was able to produce high levels of aflatoxins (>300 µg/kg) on all three kernel types under favorable conditions. Therefore, almond shipment of any kernel type should be considered susceptible to accumulating high levels of aflatoxins if *A. flavus* is present, especially during longer shipments (>10 days).

Data obtained from ocean transit studies using data loggers inside boxes of almonds recorded relative humidity levels of 44.5–61.9%, which ensure a low water activity (<0.65 a_w_) for the kernels (unpublished data). In this study, only split kernels supported the growth and aflatoxin production of *A. flavus* at 0.92 a_w_, whereas the fungus did not grow on the inshell and shelled kernels at 0.92 a_w_. Moreover, none of the three types of kernels supported the growth of *A. flavus* at 0.85 a_w_. These results suggest that maintaining water activity below 0.85 a_w_ on the kernels during transport would significantly reduce the risk of infestation and subsequent aflatoxin accumulation by *A. flavus* on all three kernel types. The current shipping conditions appear suitable to ensure safe levels of aflatoxins in almonds during ocean transit.

## 4. Conclusions

Though the optimal conditions for aflatoxin synthesis on the three types of kernels were similar, aflatoxins were produced on split almonds at a wider range of water activities. The results of this study suggested that the most effective way to limit aflatoxin production on inshell, shelled and split kernels by *A. flavus* was to maintain low water activity (<0.85 a_w_) during transit and storage. Reducing shipping and storage time also decreases the risk of aflatoxin accumulation. Future work could include different mycotoxin-producing strains of *A. flavus*.

## 5. Materials and Methods

### 5.1. Almond Samples

Three types of commercial almond kernels (inshell, shelled and split kernels of the cultivar Nonpareil) were obtained from the Almond Board of California in 2023. The inshell almonds consisted of whole kernels entirely covered by the hard outer shells. The shelled kernels consisted of whole kernels covered entirely by the seed coat only, with the hard outer shell completely removed. The split kernels consisted of whole shelled kernels cut in half, exposing the inner white flesh on the cut surface. All almond kernels had been pasteurized using commercial propylene oxide (PPO) or steam fumigation and were stored at 4 °C in plastic bags before the experiments [34]. Each sample consisted of four pieces of inshell almonds (8 g/sample), eight shelled almonds (8 g/sample) or 10 split almond kernels (4 g/sample). The kernels were placed in a single layer on 60 × 15 mm sterile Petri dishes (Corning, NY, USA).

### 5.2. Water Activity Adjustment

The initial water activity levels were 0.25, 0.26 and 0.23 a_w_ for inshell, shelled and split kernels, respectively. Autoclaved, deionized (DI) water was added to the samples using sterile pipette tips to adjust the water activity levels as follows: 800, 1500, 2000 and 2500 µL of water was added to obtain 0.85, 0.92, 0.95 and 0.98 a_w_, respectively, for inshell and shelled almonds. For the split kernels, 300, 900, 1200 and 1400 µL of water was added to obtain 0.85, 0.92, 0.95 and 0.98 a_w_, respectively. The Petri dishes were shaken to make sure that the water was equally distributed among the almonds. To maintain the water activity of the kernels, the Petri dishes were sealed (Petri-Seal Adhesive Sealing Film, CBS Scientific, USA) and incubated in closed glass jars. The water activities of the samples were verified every five days using a portable water activity meter (HygroPalm23A_w_, Rotronic, Bassersdorf, Switzerland).

### 5.3. Inoculation and Growth Measurement

*Aspergillus flavus* (NRRL 3357), an aflatoxin-producing strain isolated from moldy peanuts in the United States, was obtained from the United States Department of Agriculture Culture [35]. The fungus was allowed to grow for five days on Potato Dextrose Agar (Thermo Fisher Scientific, Waltham, MA, USA) at 27 °C in sterile Petri dishes (100 mm × 15 mm). The spore suspension was prepared in 5 mL of 0.05% Tween 80 solution and adjusted to an optical density of 0.25 at 540 nm using a spectrophotometer (Spectronic 20 Genesys, Thermo Fisher Scientific, Waltham, MA, USA). The spores were also counted with a hemocytometer (INCYTO, Chungnam-do, Republic of Korea). The suspension contained 10^5−6^ conidia/mL. All samples were point-inoculated with 15 µL of spore suspension using sterile pipette tips (Corning, NY, USA). The split kernels were inoculated on the flesh surface (no seed coat). Samples were incubated for 10, 20 or 30 days at a given combination of water activity (0.85, 0.92, 0.95 and 0.98 a_w_) and temperature (20, 27 or 35 °C). For every combination of temperature, water activity and incubation time, triplicate samples were inoculated and analyzed. After incubation for 10, 20 or 30 days, triplicate plates for each condition were removed for fungal growth and aflatoxin measurements. In addition, samples with 0.85 a_w_ were monitored for fungal growth for a 6-month observation period. Fungal growth was assessed with a 2× magnifying glass.

### 5.4. Aflatoxin Extraction and Purification

Aflatoxin was extracted at 10, 20 and 30 days of incubation from the triplicate samples. The contents of the three plates in each set of triplicate samples were combined, which resulted in 24 g of sample for inshell and shelled almonds and 12 g of sample for the split almonds. The manufacturer’s instructions were followed for the extraction of aflatoxins [36]. Briefly, the inshell and shelled samples were combined in a blender with 4.8 g of salt (NaCl) and 120 mL of methanol/water (60:40) solution, while the split samples were combined with 2.4 g of salt and 60 mL of methanol/water (60:40) solution. After blending the mixtures at high speed for 60 s, the contents of the blender were filtered into 50 mL sterile plastic centrifuge tubes using a plastic funnel and PF Filter Paper (Thermo Fisher Scientific, Waltham, MA, USA). Next, 10 mL of the filtered extract was mixed with 10 mL of DI water. The diluted extract was filtered again into a clean tube with a sterile 25 mm syringe filter (Thermo Fisher Scientific, Waltham, MA, USA). The filtrate was then purified using immunoaffinity columns (Vicam, Milford, MA, USA). First, 10 mL of the filtered diluted extract was passed through the column at a flow rate of 1 drop/second. Next, the column was washed with 10 mL of DI water followed by another wash with 10 mL of DI water. Finally, the aflatoxin was eluted with 1.0 mL of HPLC-grade methanol into glass vials (SureSTART^TM^ 2.0 mL glass vials, Thermo Fisher Scientific, Waltham, MA, USA) at a flow rate of 1 drop/second.

### 5.5. Aflatoxin Detection and Quantification

High-Performance Liquid Chromatography (Thermo Scientific Ultimate 3000 HPLC) coupled with fluorescence detector was used to detect and quantify aflatoxins. Aflatoxin analysis was carried out using fluorescence detector at 365 nm excitation and 455 nm emission. AFB_1_ and AFB_2_ were eluted through a C18, 4.6 mm × 250 mm reverse-phase column with isocratic mobile phase (HPLC grade solvents, Fisher Scientific) of water (50%), methanol (40%) and acetonitrile (10%) at a flow rate of 1 mL/min. The retention time of AFB_1_ and AFB_2_ were 8.20 and 7.12, min, respectively. Separate standard calibration curves were constructed for AFB_1_ and AFB_2_ using standard solutions (Sigma-Aldrich, Milwaukee, WI, USA) in the concentration range of 1.5 to 1000 μg/kg to quantify the levels of aflatoxin in each sample. The calibration curves for the two aflatoxins were linear with r^2^ > 0.9992 and 0.9999 for AFB_1_ and AFB_2_, respectively. The detection and quantitation limits were 1.0 and 1.5 μg/kg, respectively, under the conditions described above. Triplicate samples spiked with 18 μg/kg of AFB_1_ resulted in 99.9% recovery, which is consistent with the guidelines of 70–110% recovery rate recommended by the Joint FAO/WHO Expert Committee on Food Additives (JECFA) [37,38] while the coefficient of variation (%CV) for the measurements was 0.9%. Total aflatoxins were obtained by adding AFB_1_ and AFB_2_.

### 5.6. Statistical Analyses

The proportion of infected kernels in each Petri dish was calculated by dividing the number of infected kernels by the total number of kernels in the Petri dish. For triplicates of the same experimental condition, the proportions of infected kernels were averaged and expressed as percentages. These percentages and standard deviations were used to quantify and report fungal growth under each experimental condition.

Tukey’s multiple comparison test was used to statistically compare the differences in aflatoxin production under different combinations of temperature and water activity. The means and standard deviations of total aflatoxin production were calculated based on aflatoxin levels at 10, 20 and 30 days of incubation. A *p*-value < 0.05 was considered statistically significant.

Linear regression models were constructed to assess the statistical significance of the effects of water activity, temperature and length of incubation on total aflatoxin production using STATA IC 15 software (College Station, TX, USA). Separate models were created for each of the three kernel types to highlight the similarities and differences of these effects depending on the type of substrate. Total aflatoxin concentration (AFB_1_ + AFB_2_ expressed as µg/kg) was the dependent variable (outcome) in all three models. The independent variables (predictors) temperature and water activity were converted to categorical, because their relationships with the outcome were not linear. Days of incubation were fitted as continuous variable. Thus, the multivariable models included the categorical variable temperature (20, 27 and 35 °C), the categorical variable water activity (0.92, 0.95, 0.98 a_w_) and the continuous variable days of incubation. The water activity level 0.85 a_w_ was not included in the analysis because there was no fungal growth and aflatoxin production. The reference values in the models were 0.92 a_w_ and 20 °C. The fit of the models was ascertained by inspecting residual plots and goodness-of-fit tests.

## Figures and Tables

**Table 1:** Total aflatoxin production (AFB_1_ + AFB_2_) by A. flavus on three almond kernel types at each combination of water activity, temperature and days of incubation.

**Kernel Type**	**Days**		**Water Activity (a_w_)**											
		0.85				0.92			0.95			0.98		
		Temperature (°C)												
		20		27	35	20	27	35	20	27	35	20	27	35
Inshell	10	ND		ND	ND	ND	ND	ND	19	20	ND	14	3	ND
	20	ND		ND	ND	ND	ND	ND	4	164	256	3	372	362
	30	ND		ND	ND	ND	ND	ND	15	295	340	29	258	88
Shelled	10	ND		ND	ND	ND	ND	ND	13	321	62	209	344	188
	20	ND		ND	ND	ND	ND	ND	45	271	257	341	349	338
	30	ND		ND	ND	ND	ND	ND	133	508	463	308	292	402
Split	10	ND		ND	ND	6	6	53	4	339	243	208	327	45
	20	ND		ND	ND	4	276	26	206	297	283	305	486	349
	30	ND		ND	ND	70	453	470	482	442	279	279	334	317

ND: not detected.

**Table 2:** Multivariable linear regression model of total aflatoxin production (AFB_1_ + AFB_2_) by *A. flavus* on inshell almond kernels.

Variable	Coefficient	Lower CI *	Upper CI	*p*-Value
Temperature (°C)				
20	Reference			
27	114.15	10.21	218.11	0.033
35	106.91	2.95	210.86	0.044
Water activity (a_w_)				
0.92	Reference			
0.95	123.67	19.71	227.62	0.022
0.98	125.20	21.24	229.15	0.021
Incubation (days)	5.37	0.18	10.57	0.043
Intercept	−181.26	−322.02	−40.51	0.014

* CI: 95% confidence interval.

**Table 3:** Multivariable linear regression model of total aflatoxin production (AFB_1_ + AFB_2_) by *A. flavus* on shelled almond kernels.

Variable	Coefficient	Lower CI *	Upper CI	*p*-Value
Temperature (°C)				
20	Reference			
27	115.06	22.83	207.31	0.017
35	73.41	−18.83	165.63	0.113
Water activity (a_w_)				
0.92	Reference			
0.95	230.28	138.04	322.51	<0.001
0.98	307.86	215.61	400.09	<0.001
Incubation (days)	5.39	0.78	10.01	0.024
Intercept	−170.66	−295.55	−45.77	0.010

* CI: 95% confidence interval.

**Table 4:** Multivariable linear regression model of total aflatoxin production (AFB_1_ + AFB_2_) by *A. flavus* on split almond kernels.

Variable	Coefficient	Lower CI *	Upper CI	*p*-Value
Temperature (°C)				
20	Reference			
27	155.21	46.16	264.25	0.007
35	55.65	−53.38	164.690	0.301
Water activity (a_w_)				
0.92	Reference			
0.95	134.72	25.68	243.76	0.018
0.98	143.01	33.96	252.05	0.013
Incubation (days)	10.53	5.08	15.98	0.001
Intercept	−129.64	−277.28	17.99	0.082

* CI: 95% confidence interval.

## Data Availability

The original contributions presented in this study are included in the article, and further inquiries can be directed to the corresponding author.
